# Private sector delivery of health services in developing countries: a mixed-methods study on quality assurance in social franchises

**DOI:** 10.1186/1472-6963-13-4

**Published:** 2013-01-03

**Authors:** Karen Schlein, Anna York De La Cruz, Tisha Gopalakrishnan, Dominic Montagu

**Affiliations:** 1 The Global Health Group, University of California, San Francisco, San Francisco, CA, USA; 2 Johns Hopkins Bloomberg School of Public Health, Baltimore, MD, USA

**Keywords:** Social franchising, Private sector, Private health care, Private providers, Quality assurance, Quality, Developing countries

## Abstract

**Background:**

Across the developing world health care services are most often delivered in the private sector and social franchising has emerged, over the past decade, as an increasingly popular method of private sector health care delivery. Social franchising aims to strengthen business practices through economies of scale: branding clinics and purchasing drugs in bulk at wholesale prices. While quality is one of the established goals of social franchising, there is no published documentation of how quality levels might be set in the context of franchised private providers, nor what quality assurance measures can or should exist within social franchises. The aim of this study was to better understand the quality assurance systems currently utilized in social franchises, and to determine if there are shared standards for practice or quality outcomes that exist across programs.

**Methods:**

The study included three data sources and levels of investigation: 1) Self-reported program data; 2) Scoping telephone interviews; and 3) In-depth field interviews and clinic visits.

**Results:**

Social Franchises conceive of quality assurance not as an independent activity, but rather as a goal that is incorporated into all areas of franchise operations, including recruitment, training, monitoring of provider performance, monitoring of client experience and the provision of feedback.

**Conclusions:**

These findings are the first evidence to support the 2002 conceptual model of social franchising which proposed that the assurance of quality was one of the three core goals of all social franchises. However, while quality is important to franchise programs, quality assurance systems overall are not reflective of the evidence to-date on quality measurement or quality improvement best practices. Future research in this area is needed to better understand the details of quality assurance systems as applied in social franchise programs, the process by which quality assurance becomes a part of the organizational culture, and the components of a quality assurance system that are most correlated with improved quality of clinical care for patients.

## Background

Across the developing world healthcare services are most often delivered in the private sector. Recent systematic reviews have highlighted quality failings in both public and private care settings in developing countries [1,2] and have added power to earlier calls to standardize and assure the levels of quality offered by private providers [3]. Clinical social franchising has emerged, over the past decade, as an increasingly popular method of delivering healthcare through the private sector that has the potential to improve quality through monitoring and standardization of private, owner-operated clinics.

Social franchising aims to strengthen business practices through economies of scale: the franchisor, typically an implementing NGO with an in-country office, supports network members through branding private clinics and purchasing drugs in bulk at wholesale prices [4]. Social franchise programs often focus on reproductive health, but can include a wide variety of disease areas. The program may be a “full franchise”, meaning that the franchisor standardizes all of the products and services offered in its networked clinics, or “fractional”, in which case only certain services offered are part of the franchise program, and clinics are allowed to offer other services that are not franchised. The franchisor implements a quality assurance system that sets recruitment standards for the private providers who join the franchise, and monitors the quality of healthcare provided within the network though supervision. Franchise members agree to follow quality guidelines in order to remain in the network. Since 2006, the number of clinical health social franchises has more than doubled in low- and middle-income countries and there are now over 50 such networks operating throughout Africa, Asia and Latin America [5]. It is estimated that in 2010, over 30 million patients around the world received healthcare through a social franchise [5]. While social franchising began in the 1990s as a mechanism to deliver family planning services, it has since evolved to deliver a range of services for diseases such as malaria, pneumonia, tuberculosis, maternal child health, and HIV/AIDS [5]. The agreed upon goals of social franchising are to improve private healthcare delivery in four areas: health impact, equity, cost-effectiveness, and quality of drugs and services [6].

While quality is one of the established goals of social franchising, a systematic review of this intervention found limited evidence showing that social franchising improves quality [7]. Furthermore, there is no published documentation of how quality levels might be set in the context of franchised private providers, nor what quality assurance measures can or should exist within social franchises. A recent study has shown that in a social franchise operated by Population Services International (PSI) in Myanmar, quality management and assurance activities, broadly defined, account for nearly one-half of the overall franchise budget (unpublished analysis; Bishai D, et al.).

This study was undertaken to better understand the quality assurance systems currently used in social franchises, and to determine if there are shared standards for practice or quality outcomes across programs. The specific aims of this study were to: 1) describe frameworks by which social franchises define quality assurance within their network; 2) describe the range of quality assurance activities across social franchising programs; and 3) make recommendations for future quality assurance initiatives and research.

**Figure 1:**
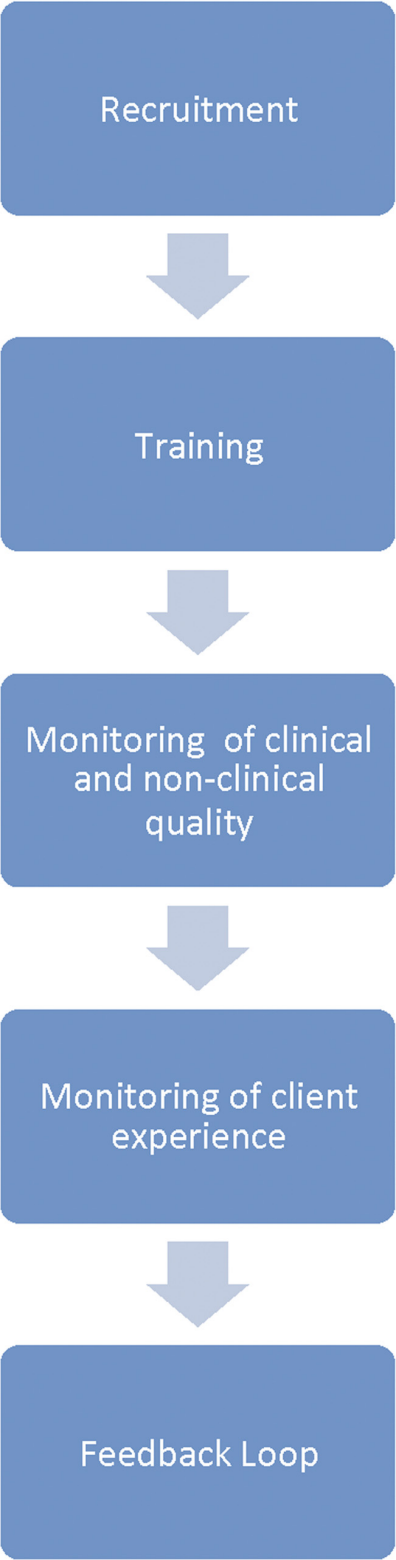
Quality assurance in social franchising framework.

In order to systematically investigate the activities related to quality assurance in social franchises, we devised a Quality Assurance in Social Franchising Framework (Figure 1) that breaks down the activities of a franchise into five general areas. We used this framework to investigate how franchises conduct quality assurance activities within each of these areas, and to group the activities into distinct phases. This investigation of quality assurance in social franchise programs was also used to select the franchise programs to be presented with a “quality assurance award” at the First Global Conference on Social Franchising in Mombasa, Kenya in November 2011.

## Methods

The study included three data sources and levels of investigation: 1) Self-reported program data; 2) Scoping telephone interviews; and 3) Site visits and in-depth field interviews with franchisor staff and franchisee clinic operators. The three-step process was intended to first identify “high performing” franchises that had comprehensive quality assurance programs that warranted in-depth on-site interviews, and to then document the quality assurance practices of those franchises with strong, highly developed quality assurance systems in place in order to draw lessons that can be applied to programs with still nascent quality controls.

The study protocol was submitted to the UCSF CHR, which determined that the study did not need to be reviewed by the full committee, and was approved to be conducted and published. Verbal informed consent was obtained from study participants before each in-depth interview. Permission was also gained from the heads of each social franchise to conduct this study.

### Program data

Fifty social franchise program managers around the world whose programs were profiled in the 2010 Social Franchising Compendium [5] were sent an English language electronic survey containing 13 questions on the quality assurance system of each franchise. These questions were designed to ask about the five components of the quality assurance framework: *recruitment, training, monitoring of clinical and non-clinical quality, monitoring of client experience and the feedback loop.*

**Figure 2:**
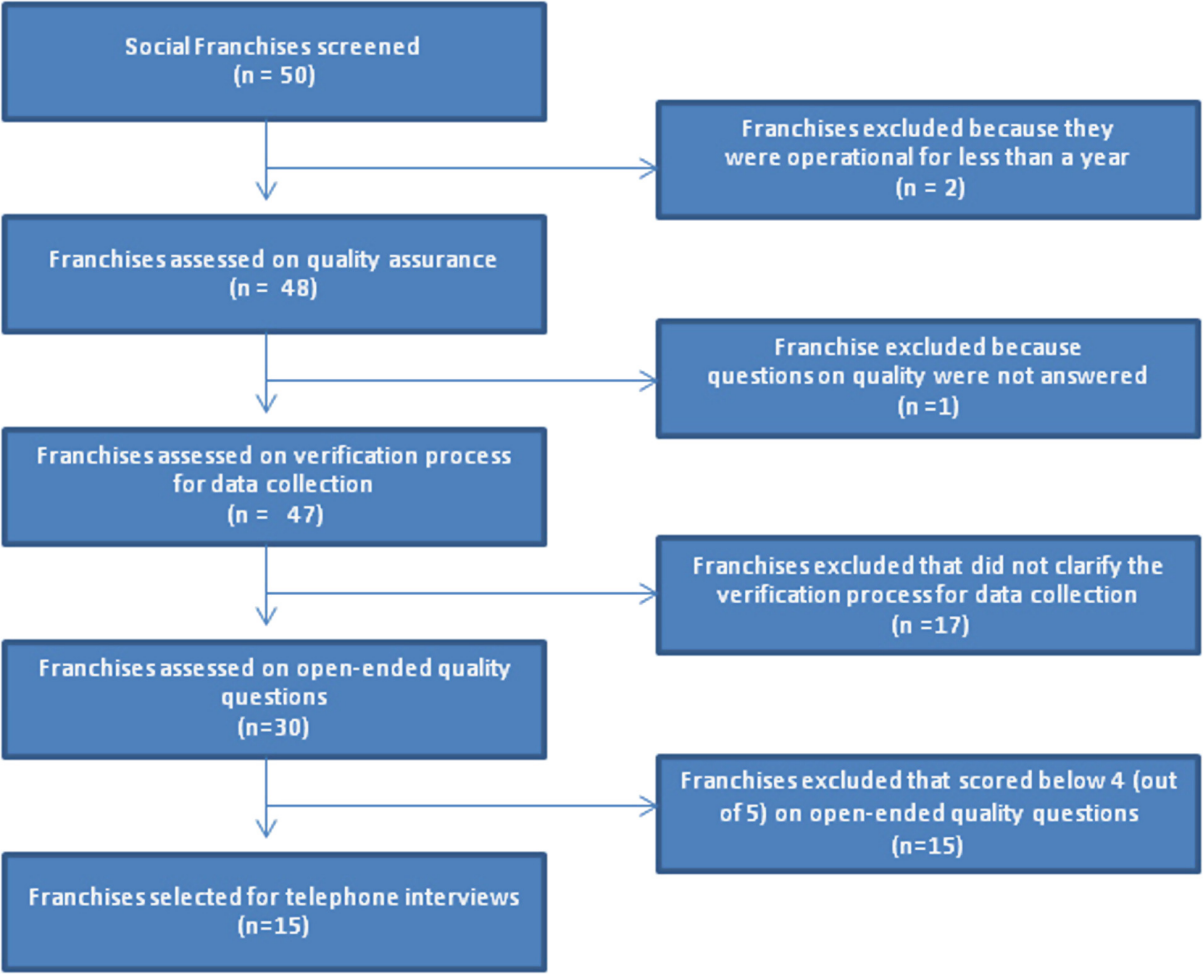
Elimination process for phone interview selection.

Starting with responses from 50 franchises, we conducted a process of elimination to select high performing franchises that would receive a scoping interview. Self reported data was solicited through open-ended questions regarding quality assurance practices and collection of program-developed quality assurance materials. The answers to open-ended questions and submitted written materials were categorized into three themes based on health quality assurance writing [8-13]: established quality standards, quality assurance systems and processes, and evidence of feedback into the system. Each program was given a score, out of a possible 5, on each of the themes. Figure 2 depicts the elimination process used to select the high performing programs, which we define as franchises that have been operational for a year or more that answered survey questions on their quality program, provided a written explanation of how quality data is verified, and scored an average 4 or 5 points (out of 5) on answers to open-ended questions based on averaged independent ratings from two researchers. The 15 franchises that scored highest according to these criteria were contacted for a telephone interview.

### Scoping telephone interviews

Our team conducted scoping interviews via phone to verify and gain additional detail on the information provided in the self-reported data. Out of the 15 interviews requested with program managers, 13 responded and agreed to an interview. We conducted 30–45 minute phone interviews with each of these organizations during the time period of June 15, 2011 to July 12, 2011. The interviewers used a structured questionnaire with ten questions that addressed the components of quality assurance in greater detail. After completion of the phone interview, we requested that the interviewees send documentation to support the phone interview, such as quality manuals, checklists, monthly/quarterly reports on quality, audit reports, and other forms or manuals used to measure or track quality. These materials were a required part of the evaluation process; franchises that did not send additional information were eliminated, as we could not verify their responses.

Based on the notes from the phone interviews and the additional documentation received, two researchers scored the franchises. Franchises were again scored in the three quality assurance categories; established quality standards, quality assurance systems and processes, and evidence of feedback into the system. Franchises were given a score of 1–5 for each category, 5 being the highest possible score for any category (15 being the highest total score). We conducted site visits at four programs to gain further insight into their quality programs, which were purposefully selected based on scores from the phone interviews, after stratification by region and the parent organization of the implementer ^1^.

### In-depth field interviews

Two researchers conducted each three-day site visit where the quality assurance systems of the franchises were assessed using a semi-structured interview guide developed based upon the Quality Assurance in Social Franchising Framework (Figure 1) and which built upon information collected during the telephone interviews. The interview guide allowed for flexibility given the differences in the four franchises and allowed interviewees to explain their role within the franchise, how they understood the meaning of quality and engaged with the quality assurance system.

Program staff at the NGO headquarters including Franchise Directors, Quality Assurance Managers, Logistics Managers, Monitoring and Evaluation Managers as well as clinic providers including doctors, nurses, midwives, and community health workers were interviewed for 30 minutes to 3 hours each. The interviews with the NGO franchise headquarter staff were longer in duration because these individuals described the full quality program. The interviews with the clinic providers were shorter, as they were describing their personal experience with quality assurance and often served as a means to verify the clinic-level implementation of quality assurance practices described by the NGO. When translation was not required by NGO staff members, the providers were interviewed by two researchers in private and the NGO staff members were requested to wait outside. This allowed researchers to verify whether provider-reported practices matched those described by NGO staff.

Coding and analysis of audio recordings and notes was completed manually by dividing the notes into themes and then grouping the corresponding themed responses into the five components of the quality framework (Figure 1). Documentation such as checklists and quality assurance plans that were provided by the franchises was similarly reviewed and categorized.

## Results

We integrated the results from all three stages of data collection (self-reported program data, scoping telephone interviews and the in-depth field interviews) in the results detailed below. The self reported program data from 50 social franchises is used to describe the overall trends in quality assurance in social franchising while the scoping telephone interviews and the in-depth field interviews provide detailed qualitative results on 13 franchises with well-developed quality programs. The 13 franchises for which we have more detailed program data include seven franchises from Asia and six from Africa. Nine are fractional franchises, and four are full franchises. The number of franchised outlets in each of the included programs ranged from 19 to 9,456, though the majority of programs had 150 or fewer franchisee outlets. Franchisors included a variety of both local and international NGOs. The four franchises selected for site visits and in-depth interviews included two in Africa and two in Asia, and also varied by size, franchisor, and included both full and fractional franchises.

### Quality assurance guiding principles

Before analyzing specific activities of a quality assurance program, we sought to understand whether or not programs based their quality assurance activities on guiding principles or a framework of quality assurance. Of the 13 high performing programs, seven franchises employ some type of guiding principles or framework by which quality assurance is understood within the organization. PSI utilizes an internally developed quality framework for their 20 social franchises, although this framework is implemented to different degrees among their franchises. This particular framework is based on five standards that are essential to quality of care: technical competence, client safety, informed choice, privacy and confidentiality, and continuity of care. For each standard, there are measurable indicators, which range from the requirement that providers be licensed and registered (technical competence) to the prohibition of provider quotas for the number of family planning acceptors or acceptors of a particular method (assuring informed choice).

Another example of a franchise network that utilizes a guiding framework is the Smiling Sun Network in Bangladesh, which defines quality assurance as “a broad concept that focuses on the entire system including suppliers and ultimate consumers of the product or service. It includes all activities designed to produce products and services of appropriate quality.” Smiling Sun has a quality management system that guides the quality assurance activities at its 9,459 outlets. The following attributes define quality of care within the network: safe, effective, customer-centered, timely, efficient and equitable. The quality assurance system is based upon the concept of a “clinic level quality circle” which means that all staff are involved and proactive in maintaining quality on a daily basis to make clinics capable and responsible for assuring, maintaining and improving quality services. The system is designed to quickly identify and resolve issues and to foster the leadership capability of clinic staff in maintaining quality standards.

### Quality assurance activities

**Figure 3:**
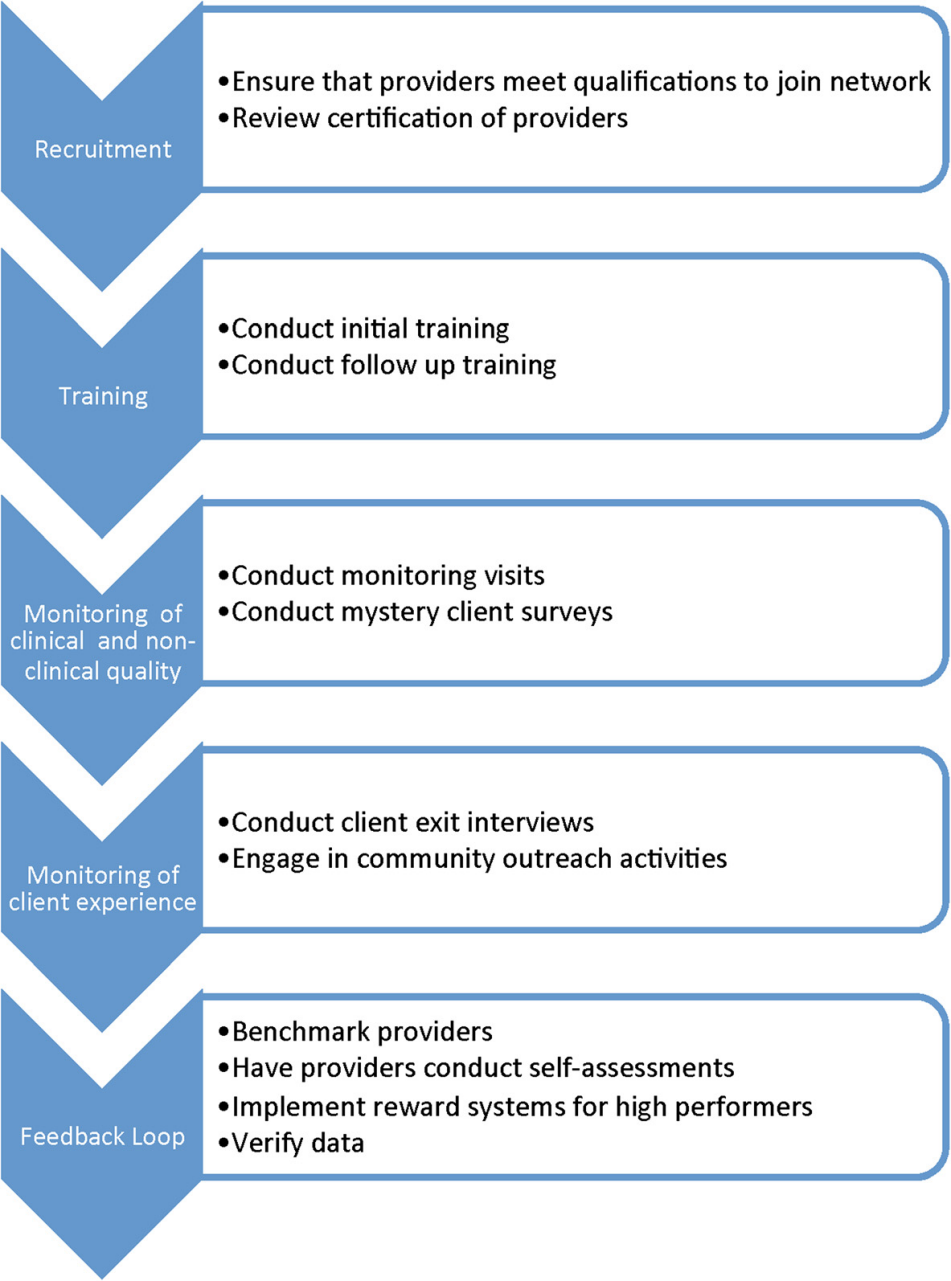
Quality assurance in social franchising framework with activities.

For each phase of the Quality Assurance Framework presented in Figure 1, social franchises reported conducting activities in which elements of quality assurance are incorporated. Figure 3 presents how quality is assured within social franchises based on the findings.

### Recruitment

Social franchises have developed recruitment and screening processes for prospective franchisees in an attempt to select providers that demonstrate the highest capability to meet the quality standards within the franchise. Based on the 13 phone interviews conducted among high performing franchises, we found that in general these franchisors recruit only providers who meet a set of standards in terms of the physical space in which they are operating. Most of the 13 franchises also require that a provider have a valid operating license. From there, the physical quality of the clinic is assessed for all selected high performing franchises. Criteria for assessment in all 13 franchises include space to provide privacy, a functioning toilet and hand washing facilities, and adequate drug storage. Many of these high performing franchisors also rate the potential clinic on its ventilation system, lighting, cleanliness of the floors and power supply and ensure that supplies to prevent cross infection, such as sterilizing equipment and disinfectants, are on hand at all times.

At Sun Quality Health in Cambodia, the recruitment process involves an initial visit where a one-page basic assessment is completed. If the minimum criteria are met, then the potential provider can officially apply to join the network. The provider is required to complete the application on her own in order to signal that she has the motivation to be part of the network. After the application is received, the recruitment team conducts a full assessment of the clinic and provider. The information from the assessment is then reviewed by a committee that sits at the NGO franchisor (Population Services International, Cambodia) who selects the top candidates. Approximately 60-70% of all providers who apply are accepted into the Sun Quality Health network.

Several of the high performing franchises also assess potential network members for personality traits and practices that franchisors consider to be predictors of delivering quality care. The Child and Family Wellness Shops franchise (CFW) in Kenya rates providers on whether they “inspire confidence” and whether they appear to be “transparent and honest.” At the Drishtee franchise in India potential providers are asked about their personal health habits; for example whether they get vaccinations and whether they eat a healthy diet. The Happy Mothers Network in Nigeria assesses each potential provider on whether she “has or is willing to establish links with the community that makes the Health Facility more accessible to low-income clients.” These personality traits do not appear to be “deal breakers” for entry into a franchise, but were considered by many franchises as an important part of the overall provider recruitment process.

### Training

Among the high performing franchises, once a provider is selected to be part of a franchise network, he or she undergoes an initial training and orientation. The length of the initial training ranges from two days to two weeks, and generally new providers are trained as a group. Provider trainees at all 13 franchisees are given pre and post tests to assess baseline and acquired knowledge, and if a minimum score is not met, franchisees are not invited to join the network. At some of these 13 franchises, franchisees who do not initially meet the minimum score are allowed to retake the training course or may be provided with individual training to help prepare them to be part of the network. The initial training is where franchisees are first introduced to the quality standards and quality assurance systems of the franchise. All high performing franchises also conduct follow up trainings for providers at various intervals where skills are refreshed and issues that have been identified in monitoring visits are reviewed in detail.

Among the 13 programs interviewed, the training programs for new franchisees provide not only classroom instruction but practicum experience where in some cases a new franchisee will work alongside more experienced clinicians who are already part of the network. At many franchises, providers are also observed conducting clinical procedures as part of a process of supportive supervision and are only allowed to begin treating patients independently once they have been observed conducting a minimum number of procedures.

At the Sun Quality Health franchise in Cambodia, all new providers complete one day of training in the classroom, one day of training on pelvic models and three days of observed practice on real clients. The providers learn about IUD insertion, removal, side effects management, counseling and screening and reporting. At the Profam franchise in Uganda, providers spend a full week practicing IUD insertions and must carry out at least five IUD insertions under supervision in order to ascertain their competency and qualify as a franchisee. At the CFW franchise in Kenya, providers attend a two-week training on clinical and financial standards before they are initiated into the network. Additionally each provider is required to spend one week working alongside an existing provider and during this practicum period is visited two times per week by the field officer. Each new franchisee is also assigned a more experienced provider as her mentor. The training at CFW does not end after the introduction period; all franchisees are required to attend a minimum of one continuing medical education training per year to remain in the network.

### Monitoring and supervision

**Figure 4:**
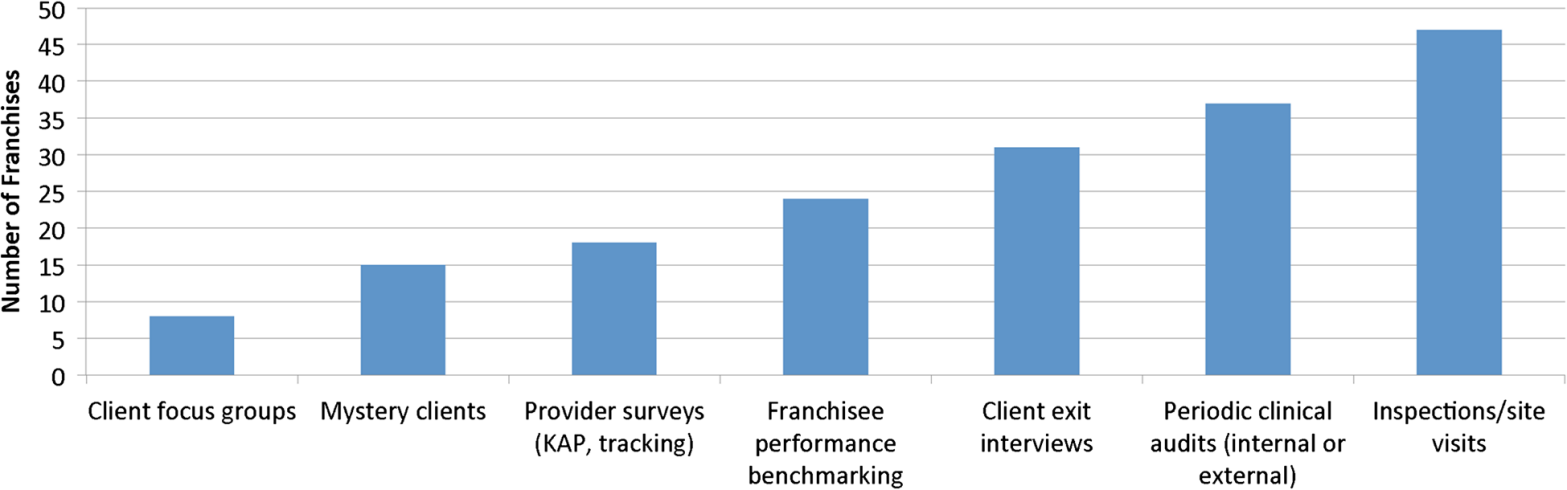
Number of franchises that conduct quality assurance activities as self-reported by 50 franchises.

Based on the self-reported data from 50 franchises, the most common quality assurance activities are regular inspections/site visits and clinical audits (Figure 4). Site visits occur regularly: 80% of franchises reported that in the past 12 months they have conducted clinical audits on 76-100% of their franchised clinics.

Ninety percent of the franchises reported having checklists which are completed by monitors during clinic visits that cover provider service delivery skills, record keeping practices, facility set up and cleanliness and also supplies (Table 1). In some franchises, the site visits are announced and in others these visits are unannounced, bringing a higher level of validity to the findings.

**Table 1:** Use of checklists to monitor quality as self-reported by 50 franchises

	**Provider service delivery skills**	**Record keeping practices**	**Facility set up and cleanliness**	**Supplies**
Yes	45	45	45	45
No	2	4	3	3
No answer	3	1	2	2

For monitoring structural quality, franchise checklists have detailed requirements. For example, at the Suraj franchise in Pakistan, a specific facility checklist is employed to assure that the clinic is a “Client Focused Center.” Within this category, items such as “*The procedure room has sufficient natural or electrical light with a back up arrangement in case of power failures*” and “*Procedure rooms are visibly clean (i.e. have no stains of blood, vomit, sputum, dust, soil, trash and spider webs on the floors, walls, windows, etc.)”* are included on the checklist. Process checklists include items for conducting specific procedures like IUD insertions. At Suraj, the IUD checklist includes items that aim to support clinical quality and minimize infections. Monitors ensure that “*the instruments are properly wrapped and that they are stored properly in a covered container.”* Additionally, we found that all franchises that we visited who are doing IUD insertions ensured that that a sterilization method is available.

Checklists are not only used to monitor structural quality, but are also used among many franchises to evaluate process quality, to ensure that clients are encouraged and supported to return to the clinic for follow up and that confidentiality and privacy were ensured. At the Happy Mother’s Network franchise in Nigeria, field monitors observe providers conducting a pre-insertion clinical exam for IUD insertion. Providers are graded on whether they “*Perform speculum exam, and locate cervix checking for any signs of cervical or vaginal problems that might preclude insertion*” and also whether the provider *“sets depth gauge on the loaded IUD inserter to the depth of the sound.”*

Franchises are not only conducting routine quality assessments, half of the 50 surveyed also reported having conducted operational research in the past year and many of the studies were related to quality assurance. These studies focused on quality topics such as quality of care delivered, client satisfaction, provider motivation and provider competence.

**Figure 5:**
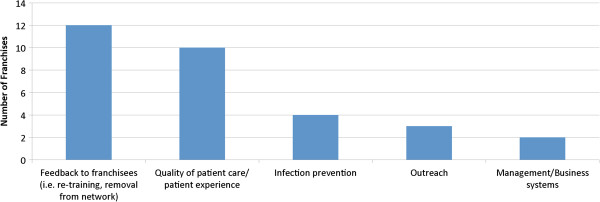
Quality assurance system results have led to improvements in these areas as self reported by 50 franchises.

The 50 franchises surveyed also reported that the quality monitoring has led to improvements in various areas of the franchise (Figure 5). Franchises used the information from quality assurance activities to implement a number of quality improvement strategies. The most common strategies reported by franchises were feedback to franchisees (i.e. re-training, removal of franchisee from network) and addressing the quality of patient care/patient experience. Other areas that were improved by the quality assurance system include infection prevention, outreach and management/business systems.

### Client experience

Overall, this investigation showed that systems to evaluate the perceived quality by patients are weaker than the systems to evaluate structural and clinical quality. While many franchises conduct client exit interviews, these surveys are not generally conducted with enough frequency to make use of the results. In the cases we observed, franchise client exit interview questionnaires contained many yes/no questions, and therefore limited data to analyze. Only seven franchises out of fifty reported conducting patient focus groups, a mechanism that may be underutilized to understand how patients perceive the quality of care.

### Feedback loop

A number of quality assurance systems include not only the guidelines and monitoring of quality, but programs to motivate and engage providers to achieve high quality. Some social franchises have established ranking systems to track the progress of franchisees and to incentivize strong performance. At some of the high performing franchises, rankings are announced publically at meetings and the top performers are awarded with recognition or with financial incentives like a new computer or a vacation package. Franchisees are engaged and responsive to such schemes and the competition is a form of motivation for them. Distribution of periodic newsletters that feature high performers is another mechanism for motivating providers. Franchises utilize a newsletter to profile high performers, motivating readers of the newsletter to increase their quality in hopes of public recognition. Public or print recognition is appealing and a particularly useful tool for assuring quality in settings where providers work independently and have limited access to training.

At the CFW franchise in Kenya, franchisees are also encouraged to join Cluster Groups, which are peer support meeting groups in which franchisees discuss complicated cases and the financial management of their clinics. Members of the Cluster Groups also contribute money into group savings, so that when a franchisee is short on cash one month, she has the option to borrow from the pool and repay later. The monthly meetings ensure that providers learn from each other and obtain feedback on their response to a particular case, helping to improve the quality of care for the network of providers.

At the Smiling Sun franchise in Bangladesh, in an effort to motivate and engage the provider in the process of assuring quality, providers are required to complete a self-assessment of their performance. After completing the self-assessment, providers develop an action plan which is a tool used to improve upon the weak areas the provider herself has identified. As part of the process, supervisors ask the providers why any weakness identified was a challenge. The Smiling Sun Clinic Provider Self Assessment guidelines provide an example and encourage probing until all barriers have been identified and the problem has been fully explored.

"Determine why you have this issue. You can use a simple “Why? Why?” exercise. For example, the issue might be that you do not have adequate space for privacy. Then ask yourself “Why?” Answer: The clinic is small. Again ask why? Why is the clinic small? “Because renovation is also costly.” Continue to ask yourself why until you feel like you have exhausted all influencing factors."

After identifying why the problem is occurring, providers are required to record possible short, medium and long-term solutions to the quality issue and also to assign a colleague to oversee the execution of the plans thus developed.

## Discussion

This investigation has limitations that are inherent in both the design and the state of social franchising implementation. While the information collected represents the most comprehensive documentation of social franchise quality programs to date, data from the initial 50 franchises was self-reported. Respondents were informed that the answers provided were going to be used to select winners of a quality assurance award which may have biased their responses and possibly led to overstating the nature of current quality assurance systems. Efforts were made to mitigate this risk through the examination of supporting documents and site visits to multiple franchise programs. The number of site visits was restricted due to budget limitations, which prohibited us from building a complete picture of the range of quality assurance programs in place around the world. Although programs reporting in all languages were encouraged, our analysis of documentation was limited to English, French, and Spanish. This covers the majority of programs and materials, but local-language quality assurance materials may exist which were not given to us, or which we were unable to properly assess. Information from the written documents collected by the 13 franchises was consistent with self-reported data. However, we could not verify “diligence of implementation” from written documentation as the checklists and forms that were sent were not populated with data from the franchises. Both the research materials and the interviews emphasized the potential positive effects of medical care. This bias on the part of programs and subsequently of this study, toward systems that assure proper procedures over systems that prevent improper procedures, is something that should be examined more carefully in future studies of clinical social franchise quality.

This study suggests that social franchises conceive of quality assurance not as an independent activity, but rather as a goal that is incorporated into all areas of franchise operations, including recruitment, training, monitoring of provider performance, monitoring client experience and the provision of feedback. Within each operational area, specific activities are conducted that are intended to assure clinical and non-clinical quality of the franchises. Franchises with strong quality assurance programs prioritize quality assurance throughout all activities in clinic operation, and market their clinics to customers as places to obtain affordable and quality healthcare in the private sector. These findings are the first evidence to support the 2002 conceptual model of social franchising which proposed that the assurance of quality was one of the three core goals of all social franchises, and a component of a ‘virtual spiral’ where higher quality would bring more clients, strengthening the brand and justifying greater emphasis on quality assurance [4]. We also found that high performing programs that have implemented a quality assurance framework, or have established and disseminated quality principles, have had success in establishing a program-wide culture of quality assurance. The frameworks or guiding principles we encountered allow all staff involved in the franchise operation to have a common language around what quality assurance means and why every individual has a role to play in assuring quality.

What is also evident from the results is that the quality assurance systems of franchises have not achieved a balance of measuring structure, process and outcome that is reflective of the research evidence base on quality assurance measurement. Overall, the systems reviewed rely heavily on structural quality evaluations: measures of the physical space including the availability of electricity and lighting and the equipment and supplies on hand; and the process for recruitment of providers into the network, and the monitoring of these providers over time. Although understandable, as structural quality is the dimension of quality easiest to measure and most correlated with the volume of clients who visit a clinic [14,15]; it is nonetheless an imperfect emphasis, as structural quality does not have a high correlation with the quality of medical advice provided by the practitioner or with health outcomes [16,17]. Process quality, the interactions between a patient and client, is more likely to explain a variance in health outcomes than is structural quality [17] and there are a number of tested methodologies for assessing process quality that would be applicable in settings where franchises operate. The use of clinical vignettes and observed simulated patients (OSP) are two methodologies that have been used successfully in low-resource and rural settings that may be practical tools for franchises to assess process quality [18-20]. Many franchises also used clinical audits as part of their quality assurance process; audits are widely used in low-resource settings [21], and can provide a measure of some clinical practices, such as what treatments are prescribed [22].

Our results also indicate that some franchises are reliant on mechanisms to evaluate quality such as client perception surveys and assessment of provider personality traits that are not necessarily validated methods of assessing or predicting quality outcomes. While we found that some franchises are investing resources to assess the client experience, it remains controversial whether patient satisfaction is related to the provision of technical quality by a healthcare provider. Patients may place high weight on the interpersonal skills of providers and the comfort of the physical space and they frequently lack the knowledge needed to assess the technical quality of healthcare [23-25]. Despite this evidence, understanding patient perception of quality can be of great value to franchises in order to appeal to existing and potential customers, and client satisfaction has been found to be a principal determinant of uptake and continued utilization of family planning services [23,24,26,27].

Rating providers on personality traits during the recruitment process was found to be standard practice at some franchises, however it is largely unknown whether specific personality traits are correlated with quality process or outcomes. Leonard et al. explore the link between the level of communication with patients and the provision of high quality clinical care in Tanzania. The authors find that clinicians who provide high diagnostic quality are not the same as the clinicians who provide high communication quality, indicating the difficultly in indentifying specific characteristics or personality traits that are predictive of a clinician who provides high quality care [28]. However the same study also found that providers who are skilled communicators are more likely to be self-motivated and not reliant on extrinsic incentives for motivation. This finding suggests that evaluating providers on their communication skills with patients could be beneficial in recruiting self-motivated providers, although not necessarily ones who are more likely to provide high quality medical care.

High performing franchises employ a range of feedback mechanisms such as publicly recognizing high performing franchisees and having franchisees conduct self-assessments that are assumed to motivate and keep providers engaged and committed. While studies on this topic conducted in low and middle-income countries (LMICs) have found positive links between feedback mechanisms and quality improvement, the study designs have tested highly specific interventions, making it difficult to draw conclusions about whether the mechanisms employed by franchises are likely to impact quality outcomes. One study on audit-based quality improvement conducted in a hospital setting in Laos found that a systematically organized education program with repeated feedback meetings improved the performance of prescribers at public hospitals, including the rational use of drugs [29]. Two systematic reviews on audit and feedback in LMIC settings have found audit with feedback to be generally quite effective in improving healthcare practices among providers [30,31].

While this study forms a basis for understanding the process by which franchises assure quality, future research in this area is needed to better understand the details of quality assurance systems as applied in social franchise programs, the process by which quality assurance becomes a part of the organizational culture, and the components of a quality assurance system that are most correlated with improved quality of clinical care for patients.

We recommend that franchises seek to implement researched alternatives to supplement the assessment of structural quality that is currently being done using one of the validated methods to evaluate process quality such as clinical vignettes or OSP, both of which have been used in low resource and rural settings. Furthermore, the evaluation of client satisfaction should be conducted on a more frequent basis, with the understanding that the results are unlikely to correlate with outcome measures of quality, but rather may provide valuable insight into adherence rates for contraception and possibly follow up visits for other disease practice areas. There are also opportunities for social franchises to conduct research within their networks to further the understanding of how specific personality traits or values are linked to the delivery of quality care and on how specific feedback mechanisms impact quality.

## Conclusions

Social Franchises conceive of quality assurance not as an independent activity, but rather as a goal that is incorporated into all areas of franchise operations, including recruitment, training, monitoring of provider performance, monitoring of client experience and the provision of feedback. Future research in this area will allow programs to better understand the details of quality assurance systems as applied in social franchise programs, the process by which quality assurance becomes a part of the organizational culture, and the components of a quality assurance system that are most correlated with improved quality of clinical care for patients. Franchise quality assurance systems overall are not reflective of the evidence to-date on quality measurement and would be greatly improved by employing methodologies to measure and improve quality that are reflective of the evidence cited in the discussion of this paper.

### Endnotes

^1^Stratifying by parent organization was important due to the high proportion of franchises run by Population Services International (PSI) and Marie Stopes International (MSI), and the resulting similarities in their own quality assurance programs.

## Abbreviations

CFW: Child Family Welfare; IUD: Intrauterine Device; LMIC: Low and Middle-Income Countries; OSP: Observed Simulated Patient; PSI: Population Services International; UCSF: University of California San Francisco.

## Competing interests

KS, DM and AYD have conducted research that has been funded by Population Services International. TG is a family member of an employee one of the social franchises surveyed for this study. Input on the study design was provided by the Social Franchising Metrics Working Group, which includes representatives from some of the franchises surveyed for this study.

## Authors’ contributions

DM conceived of the study and contributed to the manuscript preparation. KS managed refined the study design, collected the data, analyzed the results and contributed to the manuscript preparation. AYD collected the data and contributed to the manuscript preparation. TG conducted the literature review, assisted in refining the data collection tools, and collected the data. All authors read and approved the final manuscript.

## Pre-publication history

The pre-publication history for this paper can be accessed here:

http://www.biomedcentral.com/1472-6963/13/4/prepub
